# Combination therapy of TKIs and ICIs for the treatment of anaplastic thyroid carcinoma

**DOI:** 10.3389/fonc.2025.1564865

**Published:** 2025-05-23

**Authors:** Xi Zhang, Hongzhi Lu, Yuanshi Lv, Hong Gao, Hui Jin

**Affiliations:** Department of Thyroid-head & Neck Oncosurgery-1, Jilin Cancer Hospital, Changchun, China

**Keywords:** anaplastic thyroid carcinoma, TKIs, anlotinib, ICIS, sintilimab, combination therapy

## Abstract

**Objective:**

To explore the efficacy of combination therapy of tyrosine kinase inhibitor (TKIs) and immune checkpoint inhibitor (ICIs) in patients with anaplastic thyroid carcinoma (ATC).

**Method:**

This study enrolled 7 patients with ATC between June 2020 and June 2024, and conducted follow-up for a minimum of 6 months post-treatment, with the longest follow-up duration reaching 4 years. Patients received treatment with the TKI-Anlotinib and the ICI-Sintilimab, administered every 3 weeks in a treatment cycle.

**Result:**

Following treatment, among the 7 patients, the primary lesion in 3 patients who had not undergone thyroidectomy exhibited significant shrinkage, the lung metastases in 5 patients with pulmonary involvement significantly regressed, and the lateral cervical lymph node metastases in all patients demonstrated notable reduction.

**Conclusion:**

Combination therapy of TKIs and ICIs for the treatment of ATC patients can be highly effective and bringing better therapeutic outcomes and quality of life for patients.

## Introduction

Anaplastic thyroid carcinoma (ATC) is rare and highly aggressive kind of thyroid decease which accounting for approximately 1%-2% of all thyroid cancers (1, 2). ATC typically occurs in the elderly, especially in individuals over the age 60, with a slightly higher incidence in female (1). Family history of thyroid carcinoma, exposure to high dose of radiation and history of thyroid nodules or thyroid carcinoma can be the risk factors of ATC. Most patients are found to have ATC with hoarseness, difficulty swallowing and breathing, or rapidly enlarging neck mass (3, 4). Treatment for ATC is often intractable, as ATC responses poorly to traditional treatment (1).

Anlotinib, a multikinase inhibitor, targets VEGFR/FGFR associated with angiogenesis, e-Kit kinase, and PDGFR-mediated downstream signaling pathways (5, 6). Since hypoxia-induced angiogenesis facilitates the advancement of ATC, anti-angiogenic treatment plays a crucial role in the management of ATC (5). The mechanism of action of Anlotinib involves the inhibition of tumor angiogenesis and the modulation of the immune microenvironment (7–9). By targeting multiple kinases, Anlotinib disrupts signaling pathways that are crucial for tumor growth and survival (6, 10).

Sintilimab is a human immunoglobulin G4 (IgG4) monoclonal antibody, and its principle is mainly to block the interaction between the PD-1 receptor expressed by T cells and its ligands PD-L1 and PD-L2, thereby relieving the immune suppression effect and enhancing T cells’ immune surveillance and killing ability against tumors (6, 11).

In this study, we used anlotinib and sintilimab to treat patients with ATC and achieved remarkable results. This study believes that the combination of targeted therapy and immunotherapy is very useful for the treatment of ATC and deserves further research and exploration.

## Materials and methods

This is a single-center, retrospective study. Data were collected from 7 patients with ATC between June 2020 and June 2024. All 7 patients underwent combination therapy with TKI-Anlotinib and the ICI-Sintilimab and provided written informed consent for the clinical study. This retrospective investigation was approved by the Ethics Committee of Jilin Cancer Hospital.

Patients received oral administration of the TKI anlotinib at a daily dose of 12 mg for two consecutive weeks, followed by a one-week break. Every three weeks, they were administered an intravenous infusion of the PD-1 inhibitor sintilimab at a dose of 200 mg.

After 3 months of treatment with TKIs and PD-1 inhibitors, the neck lesions were evaluated by ultrasound and neck CT.

## Results

### All 7 patients received combination therapy of TKIs and ICIs

The median age of the cohort was 68 years, comprising 6 female and 1 male patient (**Table 1**). At the time of initial diagnosis, the median diameter of the primary thyroid lesion was measured at 4.0 cm. Clinically, 2 patients exhibited symptoms of hoarseness, while all 7 patients demonstrated cervical lymph node metastases, and 4 patients had developed lung metastases. Surgical intervention was pursued in 4 cases, with only 1 patient undergoing a total thyroidectomy (**Table 2**). All patients were classified as stage IVc. As of the latest follow-up, 5 patients remain alive.

**Table 1:** Patients characteristics.

Median age at first diagnosed, years (range)	68	(64-74)
Sex, n (%)		
Female	6	(85.7)
Male	1	(14.3)
Median diameter of original lesion, cm (range)	4	(1.2-5.3)
Hoarseness, n (%)		
Y	2	(28.6)
N	5	(71.4)
Lung metastasis, n (%)		
Y	4	(57.1)
N		
Cervical lymph nodes metastasis, n (%)		
Y	7	(100)
N	0	(0)
Previous surgery, n (%)	4	(57.1)
Still alive, n (%)	5	(71.4)

**Table 2:** Patients’ basic status at admission.

Patient	Age, years	Sex	Time first diagnosed	Hoarseness	Surgery
A	68	F	Jun. 2020	N	N
B	72	F	Sep. 2020	Y	N
C	74	F	Nov. 2022	N	Y
D	69	F	Oct. 2023	N	N
E	67	F	Jan. 2024	Y	Y
F	65	M	Mar. 2024	N	Y
G	64	F	May. 2024	N	Y

Among the 7 patients enrolled in the study, 3 patients did not undergo thyroidectomy. Following combined therapy, the primary lesions of ATC in 3 patients exhibited significant regression, and the diameters of lateral neck metastatic lymph nodes and pulmonary metastatic nodules were notably diminished in all patients (**Table 3**).

**Table 3:** Patients' diameter of thyroid lesion, metasized cervical lymph node and metasized lung nodule after 4 combine therapy cycle.

Patient	Diameter of lesion (before), (cm)	Diameter of lesion (after), (cm)	Shrinkage rate, (%)	Diameter of metasized cervical lymph node (before), (cm)	Diameter of metasized cervical lymph node (after), (cm)	Shrinkage rate, (%)	Diameter of metasized lung nodule (before), (cm)	Diameter of metasized lung nodule (after), (cm)	Shrinkage rate, (%)
A	5.3	5.0	6.0%	0.7	0.6	14.3%	–	–	–
B	4.8	3.2	33.3%	1.0	0.7	30.0%	–	–	–
C	3.1	–	–	2.0	0.9	55.0%	3.1	2.5	19.4%
D	4.5	2.7	40.0%	3.0	1.5	50.0%	5.0	0.7	86.0%
E	1.2	–	–	1.7	1.1	35.3%	0.8	0.3	62.5%
F	4.0	–	–	2.9	2.0	31.0%	2.3	1.2	47.8%
G	4.0	–	–	2.2	undetected	100.0%	–	–	–

Notably, Patient G, devoid of pulmonary metastases, demonstrated bone metastases in the 12th thoracic vertebra and sacral bone S1 on PET-CT. After treatment, the follow-up PET-CT showed that the hypermetabolic foci were reduced compared with before.

Patient B, also without pulmonary metastases, was found to have a newly developed 6.5 cm malignant tumor in the pancreas on PET-CT 3 years and 3 months after initiating combined therapy, raising suspicion for a metastatic lesion, although the primary origin remained elusive. After 3 years and 7 months of combined therapy, the cervical lesion began to progressively enlarge, indicating suboptimal therapeutic response and potential development of resistance. Due to tumor-induced respiratory compromise, the patient underwent tracheostomy in July 2024. Postoperative radiotherapy was administered using 6MV-X rays with VMAT (Volumetric Modulated Arc Therapy) and IGRT (Image-Guided Radiation Therapy) techniques. A total of 7 radiation fractions were delivered within 10 days, accumulating a dose of 14 Gy. Given the poor radiosensitivity of ATC and the low radiation dose delivered to tissues, radiotherapy was discontinued, and palliative care was recommended.

**Figure 1:**
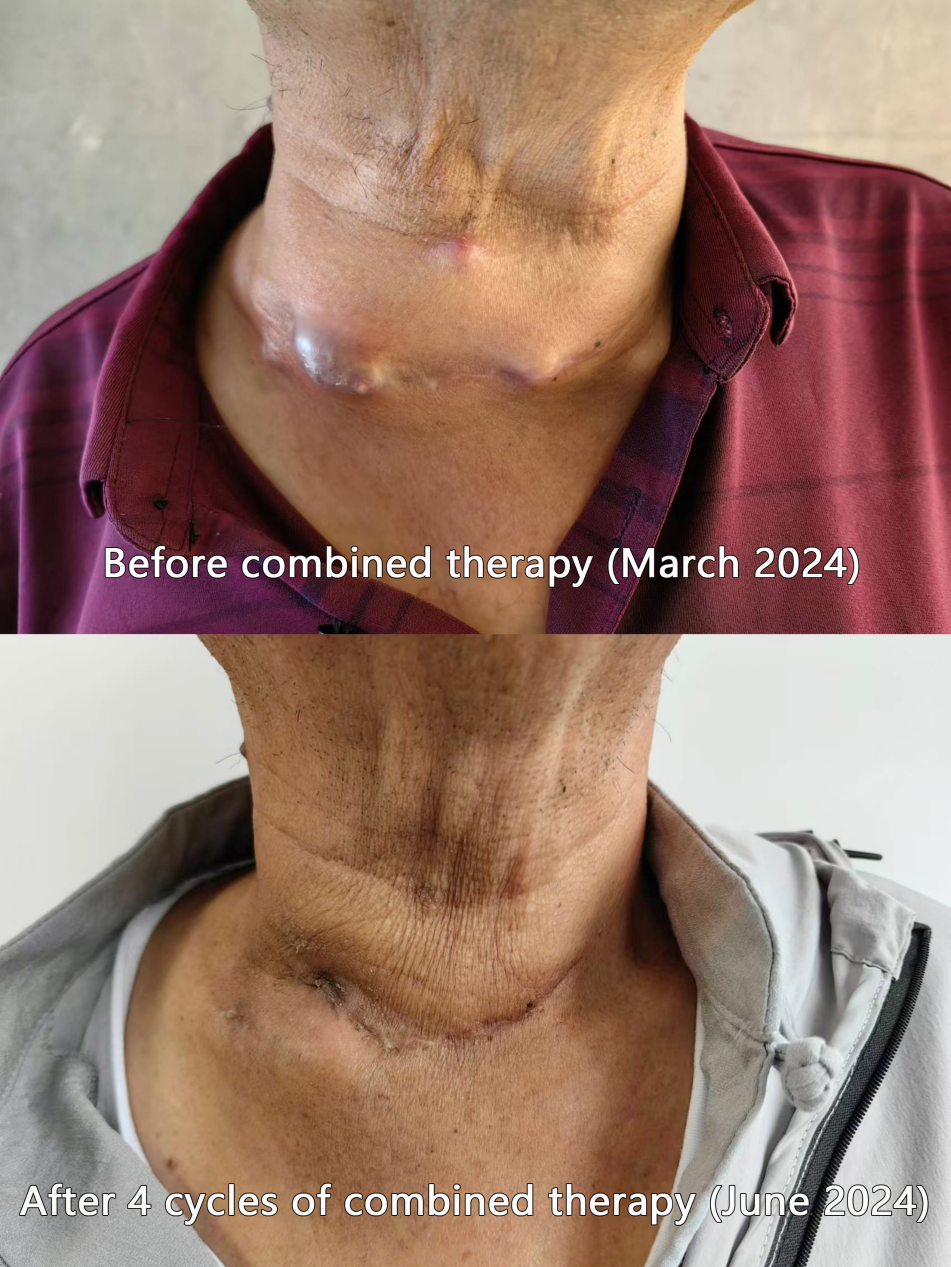
Comparison photos of Patient F before and after combined therapy. As is visible in the patient’s cervical photos, prior to the combined therapy, the tumor exhibited exophytic growth toward the anterior neck, nearly breaching the skin surface. Following the combined therapy, a marked reduction in the tumor size was observed.

As shown in **Figure 1**, Patient F exhibited a substantial mass on the anterior neck prior to the combined therapy. Following the combined therapeutic intervention, the reduction in the size of the mass was quite pronounced.

**Figure 2:**
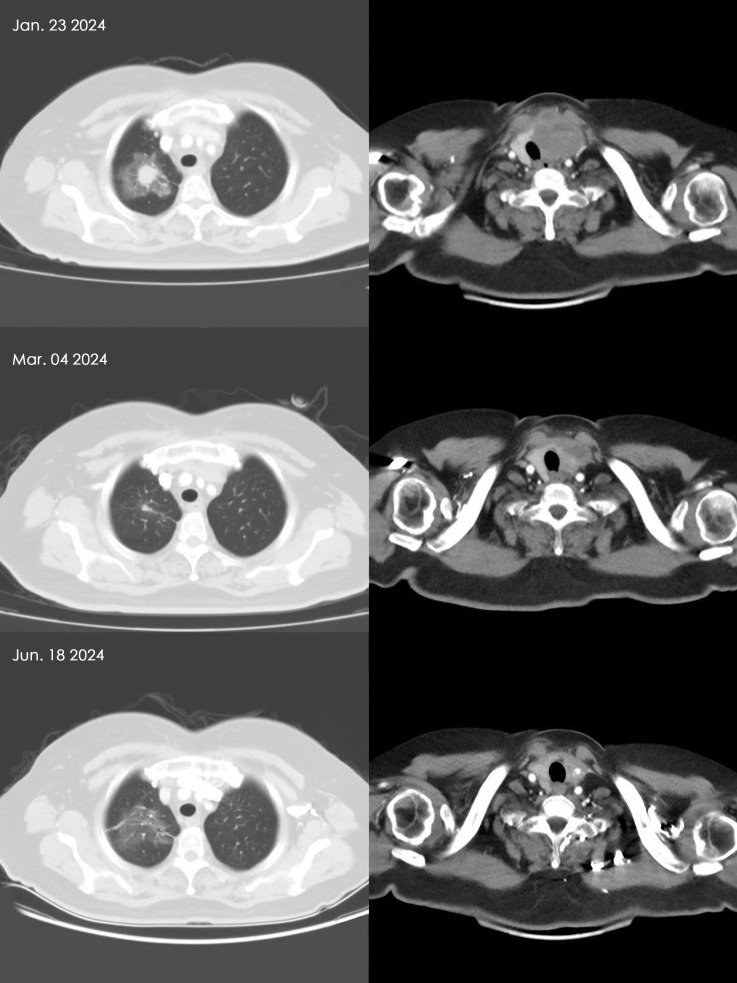
Comparison of thyroid and lung metastatic nodules in Patient D before and after combined therapy. Based on the patient’s CT scan findings, it is evident that the thyroid carcinoma and pulmonary metastatic lesions have appreciably regressed following 6 weeks and 21 weeks of combination therapy.

The CT images of the cervical and pulmonary regions for Patient D after receiving 2 cycles and 7 cycles of combined therapy are shown in **Figure 2**. There is a marked reduction in the dimensions of both the primary thyroid lesion and metastatic pulmonary lesion.

### Conduct the initial efficacy evaluation of patients after 4 combination therapy cycles

According to the RECIST v1.1 system, 1 patient achieved complete response (CR), 5 patients achieved partial response (PR), and 1 patient showed stable disease (SD). Prior to treatment, we assessed the PD-L1 expression levels of all patients. Patient A exhibited a relatively low PD-L1 expression, with insignificant reduction in the diameters of the lesion and metastatic cervical lymph nodes following treatment. Patient G had a PD-L1 expression level of 90% and attained CR after 4 combination therapy cycles.

As shown in **Table 4**, the total number of treatment cycles for patients can be observed. Initially, all patients were compliant with medical advice and adhered to regular treatment regimens. However, some patients, due to a subjective sense of well-being, were unable to maintain regular hospital visits for treatment after 10 cycles due to adverse reactions or other reasons. Among the 7 patients receiving treatment, 6 patients developed adverse reactions, with hypertension and rash being the most frequently reported.

**Table 4:** Patients’ history.

Patient	ORR after 4 cycles	PD-L1 expression, %	Treatment cycles received	Adverse event	Time of ATC progression	Time of the last follow-up	PFS, months	OS, months	Outcome
A	SD	5	19	Nausea	Jun. 2022	Sep. 2022	24	27	Discontinued treatment due to cerebral thrombosis and died of the progression of ATC.
B	PR	70	25	Hypertension	Jul. 2023	Dec.2024	34	51	Alive, after 25 cycles of combined treatment, the ATC has progressed and patient B’s condition is now poor
C	PR	30	15	Fatigue	Nov. 2023	Mar. 2024	12	16	Died of pancreatic cancer.
D	PR	90	17	Rash	–	Dec.2024	14	14	Alive, continue the combined treatment for 14 months
E	PR	50	15	Hypertension, rash	–	Dec.2024	11	11	Alive, continue the combined treatment for 11 months
F	PR	50	12	Hypertension	–	Dec.2024	9	9	Alive, continue the combined treatment for 9 months
G	CR	90	9	–	–	Dec.2024	7	7	Alive, continue the combined treatment for 7 months

Patients’ characteristics including overall response after 4 cycles, PD-L1 expression, number of treatment cycles received, adverse event, time when detected ATC progression, time of the last follow-up, PFS, OS, and outcome. Overall Response was assessed via the RACIST v1.1.

Based on the progression free survival (PFS) and overall survival (OS) of the patients, the median PFS is 12 months, and the median OS is 14 months. This study reports 2 cases of patient deaths. Patient A discontinued combination therapy due to cerebral thrombosis and was found to have disease progression of ATC after discontinuation, ultimately succumbing to ATC. Patient C abandoned combination therapy after being diagnosed with pancreatic cancer and died of pancreatic cancer after discontinuation. The discontinuation of combination therapy by these 2 patients may have led to inaccuracies in the PFS and OS values. Notably, Patient B achieved an OS of 51 months.

## Discussion

Most ATCs are found to be at IVc stage with distant metastasis and cannot be removed completely by surgical ways (12, 13). Despite several retrospective analyses indicating that a multimodal approach involving surgery along with radiotherapy and chemotherapy can positively influence the prognosis for ATC patients—such as administering postoperative adjuvant treatment with a combination of azithromycin and cisplatin, or implementing high-dose external radiotherapy after surgery—the overall prognosis remains unfavorable because of its high propensity to infiltrate adjacent tissues and metastasize distantly (14). With the development of personalized precision treatment, targeted therapies and immunotherapies have emerged as hot topic in recent years, offering renewed hope to individuals fighting with ATC.

The molecular mechanism of ATC involves various gene mutations and the imbalance of signaling pathways. The BRAF V600E mutation is the most common genetic alteration in ATC, along with mutations in TP53, PAX8, Ki-67, and other genes (15–17). BRAF V600E is the most common early driver in differentiated thyroid cancer, and 50% of ATC cases have a history or are associated with DTC, hence 10% to 50% of ATC cases harbor this mutation (18). Dabrafenib is a BRAF V600E inhibitor, while trametinib is a MEK kinase inhibitor downstream of BRAF (19, 20). The FDA approved the combination of dabrafenib and trametinib for use in BRAF V600E mutation-positive ATC in 2018.

The dysregulation of the MAPK and PI3K/AKT signaling pathways plays a crucial role in the development and progression of ATC (15, 21–23). DNA methylation exhibits high-frequency alterations in ATC, which are particularly associated with histology and correlate with gene mutations.

Anlotinib is a novel TKI developed in China that can inhibit tumor cell proliferation and angiogenesis by suppressing platelet-derived growth factor receptors, VEGFR, and others. The inhibitor acts on vascular endothelial cells through CXCL11-EGF-EGFR signaling, which can prevent the formation of microvessels even under hypoxia, interfere with the growth and proliferation of ATC cells, induce apoptosis, and inhibit migration (24).

Ruan et al. discovered through *in vitro* cell experiments that Anlotinib has anti-tumor effects on both ATC and papillary thyroid carcinoma cell lines (6). Recent studies have found that hypoxia promotes angiogenesis in ATC, and Anlotinib can effectively inhibit this phenomenon (7, 9). In addition, Anlotinib can block intercellular communication by dual inhibition of ATC and endothelial cells, and it can also reduce angiogenesis by inhibiting the epidermal growth factor receptor (EGFR) pathway. The aforementioned studies suggest that Anlotinib can exert anti-ATC effects through multiple targets, indicating its potential application prospects in ATC treatment.

As an immune checkpoint inhibitor, Sintilimab reactivates T cells’ anti-tumor activity by blocking the PD-1/PD-L1 pathway, thereby achieving the purpose of treating tumors. Currently, the main indications for Sintilimab are for the treatment of classical Hodgkin’s lymphoma, non-small cell lung cancer, and hepatocellular carcinoma (6, 11). A research indicates that the PD-L1’s combined positive scores ≥10, which demonstrated that using Sintilimab to treat patients with thyroid tumors may be effective (25).

Targeted therapy combined with immunotherapy can significantly improve the prognosis of ATC patients. It was reported that a 67-year-old ATC patient had a remarkable tumor shrinkage and an 18.3-month-sustained remission period after using Anlotinib combined with Sintilimab (26). Zichang’s report showed that TKIs combined with ICIs may be an effective way for primary squamous cell carcinoma of thyroid. The tumor volume significantly reduced and shrank after 5 courses of combined treatment of Anlotinib and Sintilimab (26). According to Laurys and Christine’s study, other types of TKIs and ICIs, such as the combination of lenvatinib and pembrolizumab, have also shown significant efficacy in ATC patients (27, 28).

The most common adverse effects of Anlotinib include hypertension, hand-foot skin reaction, oral mucositis, diarrhea, and fatigue. Adverse effects of Sintilimab include immune-mediated pneumonitis, immune-mediated hepatitis, anemia, neutropenia, hypoalbuminemia, hypertension, acute kidney injury, rash, etc. Patients are monitored for these effects, and dose adjustments may be necessary to manage toxicity.

Despite our belief in the efficacy of combination therapy, the emergence of drug resistance over time remains a formidable challenge. Once drug resistance occurs, the progression of the patient’s tumor tends to be extremely rapid, ultimately leading to mortality. Literatures indicate that the median overall survival(OS) is 3–5 months(3-4,34). In this study, Patient B, who has the longest follow-up period, has survived for four years since the detection of ATC following combination therapy. Recently, Patient B has exhibited signs of drug resistance, suggesting a poor prognosis and outcome for her disease. Although combination therapy has not completely eradicated the ATC lesion, it has at least extended the patient’s lifespan. Consequently, investigating strategies to mitigate tumor drug resistance represents a critical area for future research.

## Conclusion

Combination therapy of TKIs and ICIs for the treatment of ATC patients can be highly effective. Anlotinib can inhibit tumor cell proliferation and angiogenesis. Sintilimab reactivates T cells’ anti-tumor activity by blocking the PD-1/PD-L1 pathway. The synergistic effect of the two is even more pronounced. We hypothesize that the application of other drugs of the same type may yield comparable effect and bringing better therapeutic outcomes and quality of life for patients.

## Data Availability

The original contributions presented in the study are included in the article/supplementary material. Further inquiries can be directed to the corresponding author.
